# Patients’ Characterization of Medication, Emotions, and Incongruent Perceptions around Adherence

**DOI:** 10.3390/jpm11100975

**Published:** 2021-09-29

**Authors:** Pikuei Tu, Danielle Smith, Rachel Clark, Laura Bayzle, Rungting Tu, Cheryl Lin

**Affiliations:** 1 Policy and Organizational Management Program, Duke University, Durham, NC 27705, USA; pikuei.tu@duke.edu (P.T.); danielle.c.smith@duke.edu (D.S.); tanjaclark@gmail.com (R.C.); 2 The Link Group, Durham, NC 27713, USA; lbayzle@tlg.com; 3 College of Management, Shenzhen University, Shenzhen 518060, China

**Keywords:** compliance, qualitative, empowerment, medication acceptability, intentional nonadherence, decision, self esteem, pharmacology, rheumatology, autoimmune

## Abstract

Medication nonadherence is prevalent among patients with chronic diseases. Previous research focused on patients’ beliefs in medication or illness and applied risk-benefit analyses when reasoning their behavior. This qualitative study examined rheumatoid arthritis (RA) patients’ perceptions and feelings toward medication in parallel with attitudes about their own adherence. We conducted four 90-min focus groups and seven 60-min interviews with a diverse sample of RA patients (*n* = 27). Discussions covered dilemmas encountered, emotions, and thought process concerning medication, and included application of projective techniques. Transcripts were analyzed in NVivo-12 using a thematic coding framework through multiple rounds of deduction and categorization. Three themes emerged, each with mixed sentiments. (1) *Ambivalent feelings toward medication*: participants experienced internal conflicts as their appreciation of drugs for relief contradicted worries about side effects or “toxicity” and desire to not identify as sick, portraying medications as “best friend” and “evil”. (2) *Struggles in taking medication*: participants “hated” the burden of managing regimen and resented the reliance and embarrassment. (3) *Attitudes and behavior around adherence*: most participants self-reported high adherence yet also described frequently self-adjusting medications, displaying perception-action incongruency. Some expressed nervousness and resistance while others felt empowered when modifying dosage, which might have motivated or helped them self-justify nonadherence. Only a few who deviated from prescription discussed it with their clinicians though most participants expressed the desire to do so; open communication with providers reinforced a sense of confidence and control of their own health. Promoting personalized care with shared decision-making that empowers and supports patients in managing their long-term treatment could encourage adherence and improve overall health outcome.

## 1. Introduction

Rheumatoid arthritis (RA) is a chronic autoimmune disease characterized by inflammation and synovitis that result in joint pain, stiffness, and difficult mobility that can last for prolonged periods of time [1,2]. RA patients often experience decreased quality of life as their symptoms interfere with daily activities and impact job productivity [3,4]. Treatment of RA typically starts with one or more disease-modifying antirheumatic drugs (DMARDs) [5,6]. Biologics are added if standard-of-care DMARDs alone are ineffective [7]. As with most chronic illnesses, nonadherence is common amongst RA patients, with rates ranging from 20% to 70% [8,9,10]. In addition to an increased risk to patient safety and poorer health outcomes, nonadherence results in growing healthcare costs and the overuse of healthcare resources [11,12]. However, the effects of many interventions have been nonsignificant or short-term [13,14], and reviews found that most of the studies evaluating these programs to be low-quality or difficult to replicate [14,15].

Medication adherence is a complex behavior with hundreds of variables documented as indicators including patient-, medication-, and illness-specific and contextual factors [16,17]. It is defined as the extent to which a patient’s behavior “corresponds with agreed recommendations from a healthcare provider” [12], highlighting shared-decision making and patients’ active participation in determining their health. Compliance, commonly used interchangeably yet with a passive connotation, more specifically denotes patients following regimens including prescribed dosage, frequency, and duration [18,19]. Self-efficacy, patient-provider relationship, beliefs in medication, social support, and age were among the most frequently cited adherence factors in rheumatology [20]. Nonadherence is prevalent when patients are worried about or deterred by side-effects [21,22], false online information [23], or costs [24]. Studies often use a necessity-concerns or benefit-risk differential framework to compare facilitators and barriers to adherence quantitatively [25,26,27]. Qualitative research, which is fewer in number of publications [28,29], has described more in depth the reasoning for not complying with treatment instruction, the impact taking chronic medication has on RA patients, and the feelings of shame, uncertainty, stress, or anxiety accompanying medication-taking [30,31]. Pharmaceutics has advanced to improve the ease of consuming medication and tailored to a more personalized approach [32,33,34]. At the same time, patients’ willingness to accept regimens also relies on their perceptions toward medication and persistent behavior to complete the therapy process [16,19,35]. Few studies have concurrently considered patients’ characterization of medications beyond the risk-benefit deliberation and their interpretation of adherence to examine how these views manifest in or influence medication behavior.

Innovation and development in medicine cannot materialize their full value if patients do not take the medication. The objectives of this study, which focused on patient-related attributes, were to (a) analyze patient attitudes—including cognitive disposition and emotions—on medications in parallel with feelings regarding their own nonadherence, (b) understand triggers or motivators for self-adjusting their prescribed regimen, and (c) present a holistic view of the psychological profile of RA patients’ medication decision process via their own narratives to inform personalized care that could instill confidence in managing their chronic therapy, encouraging adherence and improving health outcomes.

## 2. Methods

### 2.1. Recruitment

We conducted four 90-min focus groups (FG 1–4) and seven 60-min individual in-depth interviews (IDI) with RA patients (*n* = 27) in the greater Raleigh-Durham area of North Carolina. Participants in the groups and interviews did not overlap. All participants were recruited through a regional research database and flyers in local rheumatology clinics. Eligibility criteria included: age 18 to 75, have been diagnosed and prescribed medication for RA for at least 6 months, and not currently or planning to become pregnant or breastfeed in the next 12 months (as these individuals may have distinctive reasons for nonadherence not applicable during regular time or to other patient population). To gather data from diverse experiences and perspectives, potential participants were contacted, screened, and selected for a purposive sample of different age, ethnicity, household income, number of years from first diagnosis, and self-reported adherence.

### 2.2. Measure

There is a number of validated scales aimed to assess patients’ adherence, such as Morisky Medication Adherence Scale (MMAS), Medication Adherence Rating Scale (MARS), Hill-Bone Scale, and Compliance Questionnaire for Rheumatology (CQR) [36,37,38,39]. These measures have been used primarily in quantitative research and clinical trials. Because this study’s objective was to appraise patients’ self-perceived adherence in relation to their actual behavior of taking medication, we posed a direct question “how would you describe how often you follow the prescription in taking all your RA medications, including the dosage, timing, and frequency as instructed?” with the intention to capture possible bias or misconception in a self-reported measure. Answers ranging from never, not often, sometimes, most of the time, and always were recorded as 1 to 5; these numbers were noted below when presenting the participants’ quotes to reflect each individual’s own view for context.

### 2.3. Procedure

We developed a semi-structured discussion guide centered around the study objectives, considering questions employed in past qualitative research and gaps identified in the literature. The guide was used for both FG and IDI to probe patients’ perceptions, experiences, and behavior related to RA medication. Discussions covered their emotions, dilemmas encountered, personal and professional relationships as a chronic patient, and thought process concerning RA disease and treatment. In one segment of each discussion, we used projective interviewing technique to gain a deeper understanding of participants’ perspectives that may not be revealed or expressed fully in direct questioning (e.g., “if your RA medication was a person, what would they be like?” instead of “what do you think of your medication?” which may render a simple answer such as “my medication is alright”) [40,41]. This technique has been widely utilized in psychology and consumer research to encourage interviewees to share their true feelings or opinions more freely rather than respond in a presumably socially acceptable or expected way [42,43]. A trained moderator facilitated the focus groups and two researchers conducted each interview. Written informed consent was obtained prior to all discussions. Recordings were transcribed by one researcher and quality checked by a second researcher.

### 2.4. Data Analysis

We performed deductive, thematic analysis [44,45] to develop a codebook. Research team members first went through all the recordings and/or transcripts individually and brainstormed the categorization of potential themes observed from the FG and IDI, initially with two general domains of patient attitudes or emotions pertaining to medication and perceptions toward non/adherence. Via multiple discourses and iterations, the team then together identified feelings toward medication itself and the action of taking medication as separate aspects, and subsequently decided on three themes most relevant to the study objectives. Two researchers independently coded all FG and IDI transcriptions using NVivo-12; disagreements in coding were discussed with and mediated by a third researcher. Representative quotes were chosen from each theme and subtheme to illustrate participants’ narratives in managing their medication. Less prominent categories considered during the coding process, such as satisfaction with provider, denial or helplessness during initial diagnosis, and social support were not presented in the Results. The study protocol was approved by Duke University Institutional Review Board.

## 3. Results

A total of 27 RA patients were interviewed in groups or individually. Participants ranged from ages 18 to 71, and time of RA diagnosis ranged from 2 to 43 years ago. The majority of participants identified as Non-Hispanic Caucasian and 25.9% were minorities. When describing how often they follow their prescriptions, three participants said sometimes, eight said most of the time, and sixteen (59.3%) said always (Table 1).

Through thematic analysis of the group and individual interviews, three themes were identified: (1) conflicting feelings toward medication, (2) concerns and complexity in medication-taking, and (3) manifestations of and attitudes toward self-adjusting medications. The first theme considered participants’ perceptions of their medications while the second depicted how they felt during or about the physical act of taking chronic medications. The third highlighted how participants viewed and conveyed their own adherence or non-compliance.

### 3.1. Ambivalent Feelings toward Medication

Many participants had negative feelings toward their medication. Some viewed it with worry and apprehension due to the many side effects of the drug; these side effects could be either personally experienced or anticipated. While appreciating the benefit medication brought in reducing or even eliminating their pain or disability, participants expressed bitterness concerning their reliance.

*“I drink gallons of water a day because I’ve got to flush that out of my system, that stuff is toxic. … my organs are probably just screaming on the inside.”*

(FG 1, SRA 5)

*“I’m a little concerned because the medication which seems to be working well, you know, can kind of damage your liver or kidneys.”*

(FG 1, SRA 5)

*“I hate prednisone. It’s like the most evil but it helps.”*

(FG 4, SRA 4)

For some participants, medication was associated with increased freedom, as the relief received from taking it enabled them to carry out more daily activities. However, sometimes the drug’s power to determine their capability seemed intimidating. This conflicting perception was vividly represented in the way participants personified their medication when prompted with the projective technique to portray their relationship with or attachment to RA medication.

*“[My medication] feels like the annoying person in junior high that’s always there … [I’m] like ‘can you leave me alone?’ … I know my medication is helping me; it’s good to have that friend.”*

(FG 4, SRA 5)

*“I guess you’d say [the medicine is my] best friend … I can’t say it makes you feel perfect, but I think without it I would feel much worse.”*

(FG 1, SRA 5)

*“It’s a Viking. So, it’s strong and fights for me and defends me. But if I’m on the bad side of my Viking, it might hurt me.”*

(FG 2, SRA 5)

*“I have a ‘date’ with (drug name) once a week. Nobody likes needles but I do love the (drug name) needle because … I promise you by, like the second day, you can feel it; I can breathe and move. It’s $3500 [for a three-month supply]—it’s like the most expensive thing in my home. But I will sell things so I can get it … [However], every time I take this needle out I’m like, oh my God, don’t let five years from now I grow an extra ear or anything.”*

(FG 1, SRA 5)

### 3.2. Struggles in Taking Medication

More than just the medication itself, participants also had repellant feelings toward the physical act of taking medication and responsibility of managing the often-complex regimens. The need to keep track of and self-administer medication was stressful and burdensome, particularly for the few who were prescribed self-injection biologics. This forced participants to identify as an ailing patient, which they struggled to accept and felt embarrassed by.

*“I really hated it because it meant that I was sick, and I was the only one that had to take medicine in my family. Even my grandmother is healthier than me.”*

(IDI 2, SRA 4)

*“I’m embarrassed when I go places, or even when our kids would have friends over … I feel like a druggie taking all this medicine when I’m 40 years old.”*

(FG 4, SRA 5)

Participants writhed to decide whether or not to take their medication and frequently considered if their desire for relief was stronger than their resentment or fears toward the medication. Some participants focused on the positives; the medicine could be a “miracle” in reducing symptoms and improving mobility, bringing back a sense of independence. Moreover, the reality of having to get injections or take pills for the rest of one’s lifetime was difficult to reconcile for participants. In the end, even participants with overwhelmingly negative feelings toward medication still described having no choice but to take it for those benefits.

*“I was able to write and function and forget about my arthritis.”*

(IDI 7, SRA 5)

*“It’s like an internal battle with myself, and I go back and forth for a couple of days before I just break down and say it’s easier just to take it.”*

(IDI 3, SRA 5)

*“I know it’s like that evil thing … You know you would be better if you could get off of it, but I cannot get off of it.”*

(FG 4, SRA 5)

### 3.3. Actions and Attitudes around Non/Adherence

#### 3.3.1. In/Congruence between Perceptions and Behavior

Unless there were serious side effects, almost all participants resigned to taking the medication. However, while some participants took the medication exactly as instructed, often noting the seriousness of the drugs, others described adjusting the dosage up or down to tailor the prescription to their own symptoms and the side effects they were experiencing at the time.

*“I take them exactly as prescribed. It’s strong medication, and if you start self-regulating, I just can’t imagine the side effects … that really scares me.”*

(FG 2, SRA 4)

*“It upset my stomach, so I started taking less and less, and trying to find that edge where it was healthy, but I wasn’t getting sick.”*

(FG 3, SRA 5)

When asked whether, when, and how they adjusted their medication, participants’ responses revealed discrepancies between their self-perception of adherence and actions. While a few participants who frequently adjusted their medication were aware of their nonadherence, most participants had reported taking their medication as prescribed most of the time or always (SRA 4 or 5).

*“Sometimes on a bad day, I will get frustrated because I feel like my medication is not working anyways, so then I don’t want the side effects as well if I take it. So, perhaps on bad days, I’m less compliant.”*

(IDI 6, SRA 3)

*“[My doctors] would prescribe that, but I would take half of that when I feel like I need it or more if I felt like I needed it.”*

(FG 1, SRA 5)

*“I just took one extra one, and it worked, so I guess I need to talk to the doctor about me having to take a little bit more than just that one pill when I am having an episode.”*

(FG 2, SRA 4)

#### 3.3.2. Emotions and Empowerment from Self-Adjusting Medication

Participants described a wide spectrum of feelings towards adjusting their own treatment regimens. Some felt anxious and risky and revealed being nervous about doctors’ reactions and potentially experiencing more symptoms or side effects, though this was not always enough to stop them from “tweaking” or experimenting. Participants also expressed worry and unsettlement when intentionally not adhering to their prescription.

*“I would say uneasy … because if I skip a dosage, or if I’m kind of playing around with it, I’m not sure what the outcome is going to be. So, it’s like a risk I’m taking.”*

(FG 3, SRA 4)

*“[I feel] like I’m a disappointment sometimes to [my care team].”*

(FG 4, SRA 4)

*“I’ll get paranoid that my joints are secretly, you know quietly getting damaged and that I’m not helping them.”*

(FG 4, SRA 5)

On the other hand, over a third of participants discussed the upside of self-adjusting medication. They felt strength in regaining control as they were able to make their own treatment decisions when they felt necessary or helpful.

*“I would say [I feel] empowered to be able to decide if I was able to take it or not whereas before I felt reliant.”*

(IDI 2, SRA 4)

*“Empowered. I mean, it’s in my hands … I feel like I’m taking charge of my own health.”*

(FG 1, SRA 5)

*“In control. It makes you feel like you’re doing a little bit of something on your own and not being told exactly, ‘You got to do this’.”*

(FG 4, SRA 5)

Compounding this was the confidence that they knew their bodies, and in particular, knew their bodies more than their clinicians did. For some participants, the longer they were on the medication and the more experience they had, the more they trusted themselves to self-adjust their dosages.

*“I make decisions based on my side-effects. If they are intolerable, I figure [my doctor] is not in my body, he doesn’t know.”*

(FG 4, SRA 4)

*“The doctor put us on a dosage, but because we know our own bodies, we know that we may feel better. So maybe we don’t need as much medication as they actually prescribed.”*

(FG 1, SRA 5)

*“I know my body, and I know by experience … At the beginning I wouldn’t do it [self-adjust my medication) and I followed the instructions of the doctor and [it] got me really in a bad spot … But then [I felt] in control because now I learned how to manage my own body, right.”*

(FG 2, SRA 5)

Only a few participants who adjusted their medications discussed it with their clinicians, though most others expressed the desire to. Open and supportive communication with their care team also made these participants feel more secure in their dosage decisions and reinforced a sense of control over their own health.

*“I think empowered and in control definitely applied later when [my doctor] started communicating with me and asking me what I wanted to do.”*

(IDI 1, SRA 4)

*“I feel in control because if [there is] something that’s not right [when I take my medication differently] I know I can always pick up the phone and call the nurse and they’ll send a message to my PA nurse, and they’ll call me back. So, I can kind of govern my own self.”*

(FG 2, SRA 5)

**Figure 1:**
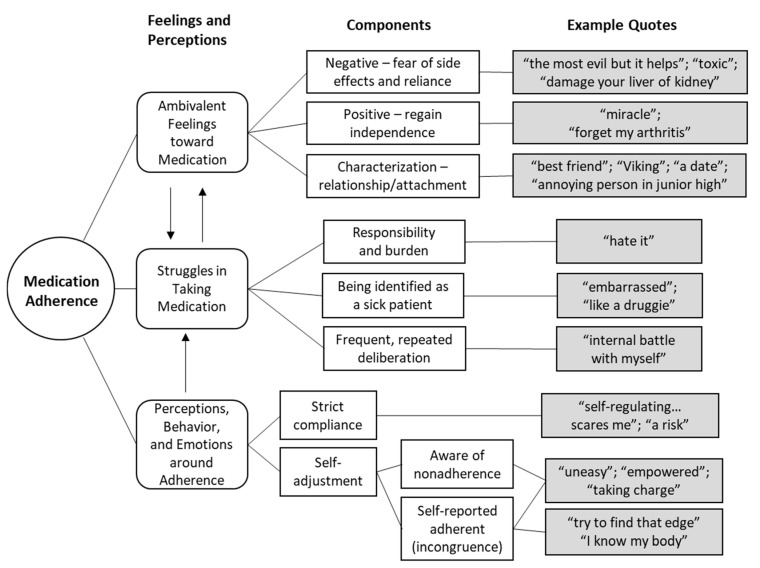
Emerged Themes of Medication Adherence from RA Patiens’ Perspective.

These narrated feelings and attitudes are synthesized in Figure 1.

## 4. Discussion

This study portrayed RA patients’ perceptions and psychological processes concerning three key components of their medication-taking behavior to help inform personalized care. The qualitative methods illustrated the paradox in their feelings toward the medication as well as the views and decisions regarding the self-adjusting of their medication. While almost all participants expressed negative feelings about the RA medication itself and about taking the medication, they still relied on it for relief and were grateful for its effects, despite the annoyance, restraint, and lack of freedom medication can cause. These findings are in line with previous studies of patients with chronic diseases and reflect the balancing act patients often wrestle with when accepting and staying compliant with a treatment regimen [46,47,48]. The projective interview technique brought out additional sentiments and struggles that were intimate to RA patients but sparsely presented in the existing literature [41,42,49]. Participants’ elaboration on their perspectives offered rich insights for evaluating medication acceptability and treatment fit beyond logistical and biological indicators.

Though discontinuation of a drug was uncommon (other than when switching medications), many participants adjusted their doses up or down if and when medications were not meeting their expectations, did not feel necessary, or had intolerable side effects. Research similarly has identified common concerns or complaints RA patients have when taking their medication [50,51,52]. Our study further pointed out the restlessness patients also have when not taking their medication, as well as the worry of other side effects from modifying the regimen, distinctive from the literature which predominantly discusses the negative view of side effects patients have when following prescriptions.

Such noncompliance was described even by participants who claimed to always be adherent. This highlights that all patients, even those with seemingly high medication acceptability or adherence rates, are at risk of intentionally deviating from prescriptions, calling for continual attention in personalized care. The evidence also helps explain the trend of overestimation in self-reporting compared to objective measures by biochemical level, pharmacy data, or monitoring devices [13,53,54,55]. The discrepancy in adherence rates or incongruency between perceptions and action in some cases may be due to different interpretations of adherence, in which the patients believed they were following the prescription, rather than due to recall bias or an attempt to appear compliant to please the provider or researchers.

Moreover, our observations exemplify how indefinite and constant the risk-benefit conflict is for patients as they had to decide if and how to adjust their dosages on a day-by-day basis depending on symptoms. Adherence programs should be tailored to address this thin balance and move towards more patient-centric interventions that not only elicit patients’ input but also understand and allow the need for modifying dosage at varied times under clinician’s guidance. Earlier studies have shown that an integrated, individualized approach is especially favorable for patients with changing needs due to unpredictable disease status such as Parkinson’s and autoimmune diseases, including RA [56,57,58,59].

Having to take chronic medication in order to function encroached on independence and self-esteem, especially if a patient wanted to reduce or avoid a medication from his or her regimen but was too dependent on it for relief to act on that desire. On the other hand, having the ability to make adjustments based on feelings and experience with the treatment or disease restored a level of self-control that they felt they had lost as their lives became overwhelmed and defined by RA and medication [60]. The feelings of empowerment and confidence may have helped participants legitimize or even incentivized their deviating behavior. Our reporting of the possible self-rationalization or self-justification and dual-view about adjusting medication suggests valuable implications to address the issue. Future research could examine the relationships between patients’ medication acceptability/tolerability, feeling of losing control, beliefs in the legitimacy or risk of modifying dosage, and changes in their adherence.

While many studies on adherence have discussed measurements and identified factors influencing patient behavior [23,46,51,52,60], we elucidated how adherence looks and feels when patients self-adjust their medication. It is important to note the complexity around the term “adherence” or “compliance” in RA research here. In contrast to other chronic diseases and though few of our participants reported it, some RA patients may be directed by their clinicians to self-adjust certain medications depending on their symptoms and side effects, blurring the line between what is considered adherent or nonadherent. Because of this complexity and variation in practice, asking RA patients whether they are adherent or taking their medications as prescribed may not sufficiently determine whether patients are accepting or appropriately using their medication. More research should be done to resolve and set a standard on how adherence is defined among RA patients. By all definitions, open discussion with patients about how they are taking their medication is crucial. Previous studies have demonstrated the benefits of patient-centered care and the association it has with empowerment and adherence [13,60,61,62,63]. Healthcare and research teams could explore ways to provide individual patients with appropriate parameters for adjusting or strategically managing treatment regimens as needed. Such practices could offer patients that sense of control and authority over their own bodies, while motivating long-term adherence.

The study is subject to limitations. The findings may not be generalizable to other patient populations given the relatively small sample size as well as the unique and fluctuating nature of RA disease activity. Additionally, there is a potential for bias in the focus groups as participants may change or withhold information in an effort to conform with the group. This limitation was mitigated by the seven in-depth interviews completed with single participants as the consistency of findings between the focus groups and interviews adds validity to our results. Future research could include interviews with physicians to discuss the appropriate adjustment of RA medications. Exploring physicians’ perspectives and understanding of how patients’ RA experiences impact adherence may also help customize care and interventions.

## 5. Conclusions

Shared decision making in healthcare suggests that physicians must understand the expectations and concerns patients have regarding their treatment [58,64,65]. Personalized care, at a higher level, requires continued attention to patients’ experiences with the disease and medication as well as their needs and preferences in therapy. This is especially important to maintain with chronic patients and over time because, concurrent with other studies [23,66], we found that as participants became more experienced with taking their medications they were more confident in adjusting their treatment regimens. Understanding how patients view and act on their own nonadherence can better inform the complexity of this behavior and ways to prevent or address it. These findings offer a more holistic view of patients’ attitudes towards adherence, considering the feelings that result from it in parallel with those that induce it. Considering the high portion of interventions with unsatisfactory results, future studies could design and assess the long-term effectiveness of programs aiming to empower patients early in the treatment process through honest discussions with clinicians in order to reduce nonadherence and achieve better disease management and outcomes.

## Figures and Tables

**Table 1:** Participant Demographics, Time of Diagnosis, and Self-Reported Adherence.

*n* = 27	*n* (%)	Mean (SD)
Gender		
Woman	21 (77.8%)	
Man	6 (22.2%)	
Age (years)		46.4 (14.56)
18–29	4 (14.8%)	
30–49	13 (48.1%)	
50–64	7 (25.9%)	
65+	3 (11.1%)	
Ethnicity		
Caucasian	20 (74.0%)	
African American	3 (11.1%)	
Hispanic	4 (14.8%)	
First diagnosed (years ago)		11.0 (8.1)
1–4	4 (14.8%)	
5–9	8 (29.6%)	
10–14	8 (29.6%)	
15–19	5 (18.5%)	
20–24	1 (3.7%)	
25+	1 (3.7%)	
Self-reported adherence		4.5 (0.7)
1 = never	0	
2 = not often	0	
3 = sometimes	3 (11.1%)	
4 = most of the time	8 (29.6%)	
5 = always	16 (59.3%)	

## Data Availability

The portion of the transcript data pertaining to this paper are available from the corresponding author for one year from publication upon reasonable request with a methodologically sound proposal.

## References

[1] Scott D.L., Wolfe F., Huizinga T.W. (2010). Rheumatoid Arthritis. Lancet.

[2] Birch J.T., Bhattacharya S. (2010). Emerging Trends in Diagnosis and Treatment of Rheumatoid Arthritis. Prim. Care Clin. Off. Pract..

[3] Ahlstrand I., Björk M., Thyberg I., Börsbo B., Falkmer T. (2012). Pain and Daily Activities in Rheumatoid Arthritis. Disabil. Rehabil..

[4] Miagoux Q., Singh V., de Mézquita D., Chaudru V., Elati M., Petit-Teixeira E., Niarakis A. (2021). Inference of an Integrative, Executable Network for Rheumatoid Arthritis Combining Data-Driven Machine Learning Approaches and a State-of-the-Art Mechanistic Disease Map. J. Pers. Med..

[5] Donahue K.E., Gartlehner G., Jonas D.E., Lux L.J., Thieda P., Jonas B.L., Hansen R.A., Morgan L.C., Lohr K.N. (2008). Systematic Review: Comparative Effectiveness and Harms of Disease-Modifying Medications for Rheumatoid Arthritis. Ann. Intern. Med..

[6] Choy E.H.S., Smith C., Doré C.J., Scott D.L. (2005). A Meta-Analysis of the Efficacy and Toxicity of Combining Disease-Modifying Anti-Rheumatic Drugs in Rheumatoid Arthritis Based on Patient Withdrawal. Rheumatology.

[7] Aletaha D., Smolen J.S. (2018). Diagnosis and Management of Rheumatoid Arthritis: A Review. JAMA.

[8] Van den Bemt B.J., van Lankveld W.G. (2007). How Can We Improve Adherence to Therapy by Patients with Rheumatoid Arthritis?. Nat. Clin. Pract. Rheumatol..

[9] Van den Bemt B.J.F., van den Hoogen F.H.J., Benraad B., Hekster Y.A., van Riel P.L.C.M., van Lankveld W. (2009). Adherence Rates and Associations with Nonadherence in Patients with Rheumatoid Arthritis Using Disease Modifying Antirheumatic Drugs. J. Rheumatol..

[10] Scheiman-Elazary A., Duan L., Shourt C., Agrawal H., Ellashof D., Cameron-Hay M., Furst D.E. (2016). The Rate of Adherence to Antiarthritis Medications and Associated Factors among Patients with Rheumatoid Arthritis: A Systematic Literature Review and Metaanalysis. J. Rheumatol..

[11] Hovstadius B., Petersson G. (2011). Non-Adherence to Drug Therapy and Drug Acquisition Costs in a National Population—A Patient-Based Register Study. BMC Health Serv. Res..

[12] Sabaté E., World Health Organization (2003). Adherence to Long-Term Therapies: Evidence for Action.

[13] Costa E., Giardini A., Savin M., Menditto E., Lehane E., Laosa O., Pecorelli S., Monaco A., Marengoni A. (2015). Interventional Tools to Improve Medication Adherence: Review of Literature. Patient Prefer. Adherence.

[14] Anderson L.J., Nuckols T.K., Coles C., Le M.M., Schnipper J.L., Shane R., Jackevicius C., Lee J., Pevnick J.M., Members of the PHARM-DC Group (2020). A Systematic Overview of Systematic Reviews Evaluating Medication Adherence Interventions. Am. J. Health Syst. Pharm..

[15] Pinho S., Cruz M., Ferreira F., Ramalho A., Sampaio R. (2021). Improving Medication Adherence in Hypertensive Patients: A Scoping Review. Prev. Med..

[16] Kvarnström K., Westerholm A., Airaksinen M., Liira H. (2021). Factors Contributing to Medication Adherence in Patients with a Chronic Condition: A Scoping Review of Qualitative Research. Pharmaceutics.

[17] Kardas P., Lewek P., Matyjaszczyk M. (2013). Determinants of Patient Adherence: A Review of Systematic Reviews. Front. Pharmacol..

[18] Hugtenburg J.G., Timmers L., Elders P.J., Vervloet M., van Dijk L. (2013). Definitions, Variants, and Causes of Nonadherence with Medication: A Challenge for Tailored Interventions. Patient Prefer. Adherence.

[19] Vrijens B., De Geest S., Hughes D.A., Przemyslaw K., Demonceau J., Ruppar T., Dobbels F., Fargher E., Morrison V., Lewek P. (2012). A New Taxonomy for Describing and Defining Adherence to Medications. Br. J. Clin. Pharmacol..

[20] Salt E., Frazier S.K. (2010). Adherence to Disease-Modifying Antirheumatic Drugs in Patients with Rheumatoid Arthritis: A Narrative Review of the Literature. Orthop. Nurs..

[21] Burmester G.R., Pope J.E. (2017). Novel Treatment Strategies in Rheumatoid Arthritis. Lancet.

[22] Villa-Hermosilla M.-C., Fernández-Carballido A., Hurtado C., Barcia E., Montejo C., Alonso M., Negro S. (2021). Sulfasalazine Microparticles Targeting Macrophages for the Treatment of Inflammatory Diseases Affecting the Synovial Cavity. Pharmaceutics.

[23] Pasma A., van ’t Spijker A., Luime J.J., Walter M.J.M., Busschbach J.J.V., Hazes J.M.W. (2015). Facilitators and Barriers to Adherence in the Initiation Phase of Disease-Modifying Antirheumatic Drug (DMARD) Use in Patients with Arthritis Who Recently Started Their First DMARD Treatment. J. Rheumatol..

[24] Harrold L.R., Briesacher B.A., Peterson D., Beard A., Madden J., Zhang F., Gurwitz J.H., Soumerai S.B. (2013). Cost-Related Medication Nonadherence in Older Rheumatoid Arthritis Patients. J. Rheumatol..

[25] Clifford S., Barber N., Horne R. (2008). Understanding Different Beliefs Held by Adherers, Unintentional Nonadherers, and Intentional Nonadherers: Application of the Necessity–Concerns Framework. J. Psychosom. Res..

[26] Zwikker H.E., van Dulmen S., den Broeder A.A., van den Bemt B.J., van den Ende C.H. (2014). Perceived Need to Take Medication Is Associated with Medication Non-Adherence in Patients with Rheumatoid Arthritis. Patient Prefer. Adherence.

[27] Kumar K., Raza K., Nightingale P., Horne R., Chapman S., Greenfield S., Gill P. (2015). Determinants of Adherence to Disease Modifying Anti-Rheumatic Drugs in White British and South Asian Patients with Rheumatoid Arthritis: A Cross Sectional Study. BMC Musculoskelet. Disord..

[28] Quandt S.A., Arcury T.A. (1997). Qualitative Methods in Arthritis Research: Overview and Data Collection. Arthritis Rheum..

[29] Kelly A., Tymms K., Fallon K., Sumpton D., Tugwell P., Tunnicliffe D., Tong A. (2021). Qualitative Research in Rheumatology: An Overview of Methods and Contributions to Practice and Policy. J. Rheumatol..

[30] Bala S.-V., Samuelson K., Hagell P., Fridlund B., Forslind K., Svensson B., Thomé B. (2017). Living with Persistent Rheumatoid Arthritis: A BARFOT Study. J. Clin. Nurs..

[31] Shaw Y., Metes I.D., Michaud K., Donohue J.M., Roberts M.S., Levesque M.C., Chang J.C. (2018). Rheumatoid Arthritis Patients’ Motivations for Accepting or Resisting Disease-Modifying Antirheumatic Drug Treatment Regimens. Arthritis Care Res..

[32] Shariff Z., Kirby D., Missaghi S., Rajabi-Siahboomi A., Maidment I. (2020). Patient-Centric Medicine Design: Key Characteristics of Oral Solid Dosage Forms That Improve Adherence and Acceptance in Older People. Pharmaceutics.

[33] Baumgartner A., Drame K., Geutjens S., Airaksinen M. (2020). Does the Polypill Improve Patient Adherence Compared to Its Individual Formulations? A Systematic Review. Pharmaceutics.

[34] Direito R., Rocha J., Sepodes B., Eduardo-Figueira M. (2021). Phenolic Compounds Impact on Rheumatoid Arthritis, Inflammatory Bowel Disease and Microbiota Modulation. Pharmaceutics.

[35] Ruiz F., Vallet T., Pensé-Lhéritier A., Aoussat A. (2017). Standardized Method to Assess Medicines’ Acceptability: Focus on Paediatric Population. J. Pharm. Pharmacol..

[36] Morisky D.E., Ang A., Krousel-Wood M., Ward H.J. (2008). Predictive Validity of A Medication Adherence Measure in an Outpatient Setting. J. Clin. Hypertens..

[37] Carl T. The Hill-Bone Scales. https://nursing.jhu.edu/faculty_research/research/projects/hill-bone/hill-bone-scales.html.

[38] Hughes L.D., Done J., Young A. (2013). A 5 Item Version of the Compliance Questionnaire for Rheumatology (CQR5) Successfully Identifies Low Adherence to DMARDs. BMC Musculoskelet. Disord..

[39] Lam W.Y., Fresco P. (2015). Medication Adherence Measures: An Overview. BioMed Res. Int..

[40] Blatt S.J. (1975). The Validity of Projective Techniques and Their Research and Clinical Contribution. J. Pers. Assess..

[41] Bell J.E. (1948). Projective Techniques: A Dynamic Approach to the Study of the Personality.

[42] Haire M. (1950). Projective Techniques in Marketing Research. J. Mark..

[43] Mesías F.J., Escribano M., Ares G., Varela P. (2018). Projective Techniques–Chapter 4. Methods in Consumer Research.

[44] Fereday J., Muir-Cochrane E. (2006). Demonstrating Rigor Using Thematic Analysis: A Hybrid Approach of Inductive and Deductive Coding and Theme Development. Int. J. Qual. Methods.

[45] Arcury T.A., Quandt S.A. (1998). Qualitative Methods in Arthritis Research: Sampling and Data Analysis. Arthritis Rheum..

[46] Müller R., Kallikorm R., Põlluste K., Lember M. (2012). Compliance with Treatment of Rheumatoid Arthritis. Rheumatol. Int..

[47] Carder P.C., Vuckovic N., Green C.A. (2003). Negotiating Medications: Patient Perceptions of Long-Term Medication Use. J. Clin. Pharm. Ther..

[48] Popa-Lisseanu M.G.G., Greisinger A., Richardson M., O’Malley K.J., Janssen N.M., Marcus D.M., Tagore J., Suarez-Almazor M.E. (2005). Determinants of Treatment Adherence in Ethnically Diverse, Economically Disadvantaged Patients with Rheumatic Disease. J. Rheumatol..

[49] Jones B., Hunt A., Hewlett S., Harcourt D., Dures E. (2021). Rheumatology Patients’ Perceptions of Patient Activation and the Patient Activation Measure: A Qualitative Interview Study. Musculoskeletal Care.

[50] National Collaborating Centre for Primary Care (2009). Patients’ experience of medicine-taking. Medicines Adherence: Involving Patients in Decisions About Prescribed Medicines and Supporting Adherence.

[51] Li L., Cui Y., Yin R., Chen S., Zhao Q., Chen H., Shen B. (2017). Medication Adherence Has an Impact on Disease Activity in Rheumatoid Arthritis: A Systematic Review and Meta-Analysis. Patient Prefer. Adherence.

[52] Salt E., Frazier S.K. (2011). Predictors of Medication Adherence in Patients with Rheumatoid Arthritis. Drug Dev. Res..

[53] Shi L., Liu J., Koleva Y., Fonseca V., Kalsekar A., Pawaskar M. (2010). Concordance of Adherence Measurement Using Self-Reported Adherence Questionnaires and Medication Monitoring Devices. Pharmacoeconomics.

[54] Foley L., Larkin J., Lombard-Vance R., Murphy A.W., Hynes L., Galvin E., Molloy G.J. (2021). Prevalence and Predictors of Medication Non-Adherence among People Living with Multimorbidity: A Systematic Review and Meta-Analysis. BMJ Open.

[55] Varallo G., Scarpina F., Giusti E.M., Suso-Ribera C., Cattivelli R., Guerrini Usubini A., Capodaglio P., Castelnuovo G. (2021). The Role of Pain Catastrophizing and Pain Acceptance in Performance-Based and Self-Reported Physical Functioning in Individuals with Fibromyalgia and Obesity. J. Pers. Med..

[56] Van Munster M., Stümpel J., Thieken F., Pedrosa D.J., Antonini A., Côté D., Fabbri M., Ferreira J.J., Růžička E., Grimes D. (2021). Moving towards Integrated and Personalized Care in Parkinson’s Disease: A Framework Proposal for Training Parkinson Nurses. J. Pers. Med..

[57] Metta V., Batzu L., Leta V., Trivedi D., Powdleska A., Mridula K.R., Kukle P., Goyal V., Borgohain R., Chung-Faye G. (2021). Parkinson’s Disease: Personalized Pathway of Care for Device-Aided Therapies (DAT) and the Role of Continuous Objective Monitoring (COM) Using Wearable Sensors. J. Pers. Med..

[58] Taylor P.C., Betteridge N., Brown T.M., Woolcott J., Kivitz A.J., Zerbini C., Whalley D., Olayinka-Amao O., Chen C., Dahl P. (2020). Treatment Mode Preferences in Rheumatoid Arthritis: Moving Toward Shared Decision-Making. Patient Prefer. Adherence.

[59] De Belvis A.G., Pellegrino R., Castagna C., Morsella A., Pastorino R., Boccia S. (2021). Success Factors and Barriers in Combining Personalized Medicine and Patient Centered Care in Breast Cancer. Results from a Systematic Review and Proposal of Conceptual Framework. J. Pers. Med..

[60] Lin C., Tu R., Bier B., Tu P. (2021). Uncovering the Imprints of Chronic Disease on Patients’ Lives and Self-Perceptions. J. Pers. Med..

[61] Castro E.M., Van Regenmortel T., Vanhaecht K., Sermeus W., Van Hecke A. (2016). Patient Empowerment, Patient Participation and Patient-Centeredness in Hospital Care: A Concept Analysis Based on a Literature Review. Patient Educ. Couns..

[62] Bartkeviciute B., Lesauskaite V., Riklikiene O. (2021). Individualized Health Care for Older Diabetes Patients from the Perspective of Health Professionals and Service Consumers. J. Pers. Med..

[63] Menditto E., Orlando V., De Rosa G., Minghetti P., Musazzi U.M., Cahir C., Kurczewska-Michalak M., Kardas P., Costa E., Sousa Lobo J.M. (2020). Patient Centric Pharmaceutical Drug Product Design—The Impact on Medication Adherence. Pharmaceutics.

[64] Taylor P., Manger B., Alvaro-Gracia J., Johnstone R., Gomez-Reino J., Eberhardt E., Wolfe F., Schwartzman S., Furfaro N., Kavanaugh A. (2010). Patient Perceptions Concerning Pain Management in the Treatment of Rheumatoid Arthritis. J. Int. Med. Res..

[65] Elwyn G., Frosch D., Thomson R., Joseph-Williams N., Lloyd A., Kinnersley P., Cording E., Tomson D., Dodd C., Rollnick S. (2012). Shared Decision Making: A Model for Clinical Practice. J. Gen. Intern. Med..

[66] Mathews A.L., Coleska A., Burns P.B., Chung K.C. (2016). The Evolution of Patient Decision-Making Regarding Medical Treatment of Rheumatoid Arthritis. Arthritis Care Res..

